# Pleural Mesothelioma in New Caledonia: Associations with Environmental Risk Factors

**DOI:** 10.1289/ehp.1002862

**Published:** 2010-12-30

**Authors:** Francine Baumann, Pierre Maurizot, Morgan Mangeas, Jean-Paul Ambrosi, Jeroen Douwes, Bernard Robineau

**Affiliations:** 1 Université de la Nouvelle-Calédonie, Equipe de Recherche en Informatique et Mathématiques, Noumea, New Caledonia; 2 Bureau de la Recherche Géologique et Minière, Noumea, New Caledonia; 3 Institut de Recherche pour le Développement, US140 Espace, Noumea, New Caledonia; 4 Centre Européen de Recherche et d’Enseignement en Géosciences de l’Environnement, Aix Marseille University, CNRS, Aix-en-Provence, France; 5 Centre for Public Health Research, Massey University, Wellington, New Zealand; 6 Service Géologique, Direction des Mines et de l’Energie de Nouvelle-Calédonie, Noumea, New Caledonia

**Keywords:** antigorite, asbestos, cluster analysis, ecological study, environment, mesothelioma, New Caledonia, serpentinite

## Abstract

**Background:**

High incidences of malignant mesothelioma (MM) have been observed in New Caledonia. Previous work has shown an association between MM and soil containing serpentinite.

**Objectives:**

We studied the spatial and temporal variation of MM and its association with environmental factors.

**Methods:**

We investigated the 109 MM cases recorded in the Cancer Registry of New Caledonia between 1984 and 2008 and performed spatial, temporal, and space–time cluster analyses. We conducted an ecological analysis involving 100 tribes over a large area including those with the highest incidence rates. Associations with environmental factors were assessed using logistic and Poisson regression analyses.

**Results:**

The highest incidence was observed in the Houaïlou area with a world age-standardized rate of 128.7 per 100,000 person-years [95% confidence interval (CI), 70.41–137.84]. A significant spatial cluster grouped 18 tribes (31 observed cases vs. 8 expected cases; *p* = 0.001), but no significant temporal clusters were identified. The ecological analyses identified serpentinite on roads as the greatest environmental risk factor (odds ratio = 495.0; 95% CI, 46.2–4679.7; multivariate incidence rate ratio = 13.0; 95% CI, 10.2–16.6). The risk increased with serpentinite surface, proximity to serpentinite quarries and distance to the peridotite massif. The association with serpentines was stronger than with amphiboles. Living on a slope and close to dense vegetation appeared protective. The use of whitewash, previously suggested to be a risk factor, was not associated with MM incidence.

**Conclusions:**

Presence of serpentinite on roads is a major environmental risk factor for mesothelioma in New Caledonia.

Malignant mesothelioma (MM) is a rare and fatal tumor of the pleura, with a worldwide annual incidence of one or two cases per million inhabitants. The incidence has increased since the 1950s among men in regions where asbestos was mined or industrially used (MacDonald and MacDonald 1986). Inhalation of asbestos fibers is the only established causal factor for MM (Merler and Chellini 1992). MM may also occur after exposure to erionite fibers (Baris et al. 1979), or fluoroedenite fibers (Putzu et al. 2006). The risk of MM increases with level and duration of exposure (Cugell and Kamp 2004), with a median latency period of 30–40 years (Montanaro et al. 2003). Incidence rates have been reported to be 1.2–9 times higher among men than among women because of occupational exposure (Ross and McDonald 1995).

MM has been associated with domestic exposure in family members of asbestos workers (Ferrante et al. 2007) and with environmental exposure (Hillerdal 1999; Maule et al. 2007) related to asbestos factories, mines, or naturally occurring asbestos in Greece (Constantopoulos et al. 1987), Cyprus (McConnochie et al. 1987), Turkey (Carbone et al. 2007), Corsica (Rey et al. 1993), Italy (Magnani et al. 2001), and the United States [California (Pan et al. 2005)].

There are two main families of asbestos: amphiboles (tremolite, actinolite, crocidolite) and serpentines (chrysotile, antigorite, lizardite). Asbestos fibers vary in length and shape: chrysotile has long, flexible fibers, whereas amphiboles fibers are brittle. Although all types of asbestos fibers can be associated with MM, the highest risks have been reported for amphiboles (Hodgson and Darnton 2000). Health problems associated with exposure to airborne asbestos particles have resulted in severe restrictions on the use of asbestos from the 1980s. In natural environments, asbestos is commonly associated with ultramafic rocks (rich in iron and magnesium minerals), mostly peridotites, of which hydration produces serpentinites (Schreier 1989).

A third of the main island of New Caledonia, called the Grande Terre, consists of ultramafic rocks, mainly peridotites and serpentinites (Birrell and Wright 1945). Three geological areas may contain asbestos: the Boghen unit, the Peridotite massifs, and the Northern Caledonian metamorphic complex (Figure 1). The territory is divided into 33 communes that are administrative areas centered in a city or village. These communes include a few Melanesian tribes or several dozen. Nickel mining and smelting is the main economic activity of the country, and nickel ores have been shown to contain asbestos (Langer et al. 1980).

Very high incidences of MM and lung cancer have been observed in New Caledonia. For the 1996–2005 period, the age-standardized incidence rate (ASR) of MM was 4.6 per 100,000 person-years among men and 3.1 among women; lung ASR in Melanesians was 92.8 per 100,000 person-years among men and 27.8 among women (Baumann and Rougier 2005). A previous study showed an association between MM and the use of a whitewash made with local soils, called “Pö” (Goldberg et al. 1995). Analysis of some Pö samples confirmed that this whitewash could contain tremolite.

We conducted a case–control study of MM using the 68 cases recorded in the Cancer Registry of New Caledonia for the 1984–2002 period (Baumann et al. 2007b). The world ASR of MM was 7.52 per 100,000 persons among Melanesians and 0.57 among Caucasians. The early onset of the disease, beginning at the age of 30 years, and the sex ratio of 1.03, suggested a nonoccupational cause for Melanesians. Thirty percent of the cases lived in Houaïlou, an area where mining activity was important during the 1960s–1970s. Geographical analysis demonstrated a significant association between soil containing serpentinite and MM.

In this article, we present the results of spatial, temporal, and space–time cluster analyses on all MM cases recorded in New Caledonia between 1984 and 2008, and an ecological analysis conducted in a specific area that included the places with the highest MM incidences. The main objectives of this ecological study were to analyze the clusters of MM, and the associations between MM and exposure to natural sources of asbestos.

## Materials and Methods

We identified MM cases from the Cancer Registry of New Caledonia. Eligible cases were malignant pleural mesotheliomas that were histologically confirmed and diagnosed from 1984 to 2008.

We conducted epidemiological investigations on all 109 eligible MM cases. Using a standard questionnaire, we conducted interviews with the living cases (12%) or two of their closest relatives to collect demographic information: sex; date of birth; race/ethnicity; date of diagnosis; residential histories, including location and description of each location; occupational history; and school history. Of the cases interviewed, we excluded five because they had lived in New Caledonia for < 20 years before the date of diagnosis. All participants gave their informed consent. These investigations were conducted within the framework of the cancer registry and were approved by the French and the local authorities.

**Figure 1:**
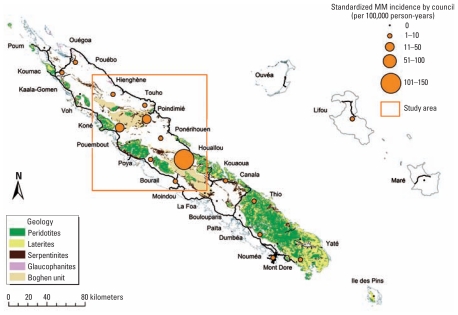
Map of New Caledonia, asbestos-bearing geological units, main roads, standardized incidence of MM by commune (residence 30 years before diagnosis), 1984–2008, and study area.

We identified a study area that included the communes with the highest MM incidences and the three geological units that may contain asbestos (Figure 1). We conducted an ecological analysis of the 100 tribes living in this area by comparing environmental exposure between tribes with and without MM cases. Environmental determinants, such as presence of serpentinite on roads, mining activity in close proximity, and vegetation cover, were assessed at the tribal level and will be described briefly below.

### Environmental and geological investigations

To locate the known bodies of ultramafic rocks and serpentinite and peridotite massifs—the potential sources of natural asbestos—we used a digital version of the BD SIGEOL 1/50,000 geological maps (Gouvernement de la Nouvelle-Calédonie 2010). We conducted geological and environmental investigations in the environment of each of the 100 tribes. After our first investigations (Baumann et al. 2007a, 2008), a census of serpentinite quarries was carried out by the service of Mines and Quarries (Gouvernement de la Nouvelle-Calédonie 2010), which we also used. Serpentinite was widely used to cover roads and may thus be a potential source of exposure. The territorial and the provincial roads, which are the most used, began to be sealed in the 1970s in the south and in the 1990s in the north.

In each tribe, we questioned elders about the material used to whitewash the houses, the history of this practice, and the localities of the quarries of Pö. We collected samples of indoor and outdoor Pö on the walls of existing whitewashed dwellings and in the quarries. We detected three categories of Pö: tremolitic (containing asbestos fibers), nontremolitic, and partly tremolitic (some families used tremolitic Pö; others used a material without fibers). We collected samples of all natural materials suspected to contain asbestos in the close vicinity of the tribe including outcrops, road cuts, and quarries.

We carried out mineralogical analyses on the 486 samples collected to assess the presence of asbestos. We first analyzed fibers using polarized light microscopy (PLM). Where required, we used CM20 200 kV analytical transmission electron microscope X-ray powder diffraction and transmission electron microscopy (Philips, Amsterdam, the Netherlands) coupled with an EDAX energy-dispersive X-ray spectrometer (Ametek, Mahwah, NJ, USA) to determine the nature of the asbestos. For cost reasons, we analyzed only 94 samples using both approaches. Of the 392 remaining samples, 97 were negative, and the PLM was sufficiently reliable to determine the nature of the asbestos for the remaining samples.

We constructed an index of mining activity influence as follows:


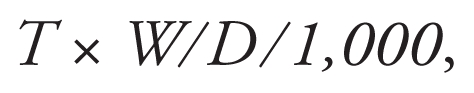


where *T* is the tonnage of nickel ore extracted between 1940 and 1980; *W* is a coefficient depending on the orientation of the mining massif with respect to the tribe and the prevailing wind; and *D* is the distance between the center of the tribe and the nearest massif.

We integrated all data into the geographic information system (GIS) ArcGIS (version 9.02; ESRI, Redlands, CA, USA). Mean slope and mean curvature over 1 km^2^ around the center of the tribe, surfaces areas, and all distances were automatically calculated using ArcGIS on a 50-m cell-size digital terrain model (MNT 50, Gouvernement de la Nouvelle-Calédonie, Nouméa, Nouvelle-Calédonie).

### Statistical analyses

We calculated the 95% confidence intervals (CIs) of incidences on the assumption that the number of cases observed followed a Poisson distribution (after testing the compliance with a Poisson distribution). Because of the mean latency of 30 years—minimum 20 years—we used two methods to study the geographical distribution of MM cases. The first involved the longest place of residence, provided that the subject had lived there for at least 20 years before diagnosis. The second method involved the place where the subject was residing in the 30th year before the date of diagnosis. For each commune and each tribe, we calculated crude and world ASRs of MM with their 95% CI by both analysis methods. We used the population data from the 1983, 1989, 1996, and 2004 census.

We performed a spatial, temporal, and space–time disease clustering analysis to detect possible areas or periods associated with a significantly higher risk of MM. We used the Kulldorff’s spatial scan statistical method that adopts maximum likelihood techniques to test for the presence of clusters and identify their location. The spatial scan analysis was implemented in SaTScan software (version 8.2.1; SaTScan, Boston, MA, USA). Sex and age were considered as covariates. We evaluated clusters using 999 Monte Carlo simulations.

For the univariate and multivariate analyses, we grouped each tribe with < 50 inhabitants (1989 census) with the closest tribe, provided that it was located in the same valley or no further than 5 km away from the coast line. We analyzed the associations between exposure estimates and MM by two methods: we first compared tribes with and without MM cases and calculated odds ratios (ORs), then Poisson regression was applied to calculate incidence rate ratios (IRR). We used the first approach because it is statistically more effective in detecting a significant factor, and the second to take into account the extent of MM incidence and to estimate the linear influence of the factor studied on this incidence. To show how the risk of incidence changed with the categorized variables, ASRs are reported using the lowest exposure category as reference groups for risk factors, and the highest exposure category for protective factors. Continuous variables were first categorized by tertiles. If two contiguous classes were comparable, the variable was then categorized into two classes. To enhance the strength of the results, we carried out multivariate analyses on bicategorized dependent variables.

We compared means with a *t* test, Welch variant when the Bartlett’s test for equal variances was significant, and Kruskal-Wallis test when more than two groups were compared. Percentages were compared with a Pearson chi-square or a Fisher’s exact test when numbers were small. The statistical tests applied to compare incidence rates are based on the convergence of the Poisson distribution toward the normal distribution. Multivariate analyses were conducted using backward stepwise logistic and Poisson regressions, including all the variables having a *p*-value < 0.25 in the univariate analysis. All interactions were tested by using the likelihood-ratio test. The most significant interactions were included in the multivariate logistic model. When two variables were strongly correlated (*r* > 0.7), only one of them was included in the multiple Poisson regression model. ORs and IRR are given with their 95% CIs. All reported *p*-values are two-tailed.

The statistical calculations were performed with R (R Foundation for Statistical Computing, Vienna, Austria) and Stata SE software (version 8.0; StataCorp, College Station, Texas, USA).

## Results

Among the 104 MM cases, 49 were men and 55 women. Ninety-one (87%) were Melanesians, eight (8%) Caucasians, and five (5%) Tahitians. Age varied from 31 to 81 years, with a mean of 60.0 years, comparable in both sexes. Mean age was higher for Caucasian men (71.9 years of age) than for Melanesian men (60.3 years of age). The sex ratio (male:female) was higher for non-Melanesians (2.25) compared with Melanesians (0.784). See Supplemental Material, Table 1 (doi:10.1289/ehp.1002862) for a more detailed overview of population characteristics.

A large proportion of men had many different jobs for very short periods (i.e., a few months). Therefore, detailed analyses of occupation exposure were not feasible.

Both methods used to analyze place of residence showed no significant difference [Supplemental Material, Figure 1 (doi:10.1289/ehp.1002862)]. The results are presented according to the place of residence 30 years before the date of diagnosis.

### MM incidence

Crude and standardized MM incidences were high in three communes (Figure 1). Compared with the main city of Noumea (ASR = 0.64; 95% CI, 0.13–1.88), we found that the MM incidence was 200 times higher in Houaïlou (ASR = 128.66; 95% CI, 70.41–137.84), 40 times higher in Koné (ASR = 25.46; 95% CI, 14.25–41.98), and 25 times higher in Poindimié (ASR = 15.37; 95% CI, 3.17–44.92). The study area included 7 of the 10 communes with the highest MM incidence.

The number of cases by tribe varied from 0 to 7. The logarithm of ASR showed a bimodal distribution, that is, 62 tribes had no incidence, and the 38 others presented a symmetric distribution centered on the median of 4. The chi-square test of compliance of the observed distribution among these 38 tribes with a Poisson distribution did not show any significant deviation.

Seventy-eight MM cases lived in the study area 30 years before diagnosis. One of them had never lived in a tribe and was excluded from the ecological analysis. The other 77 cases were Melanesians, except for one Polynesian. The sex ratio M:F of < 1 and the same mean age at diagnosis for both sexes (< 60 years of age) confirmed the high probability of an environmental causal exposure.

### Cluster analyses

**Figure 2:**
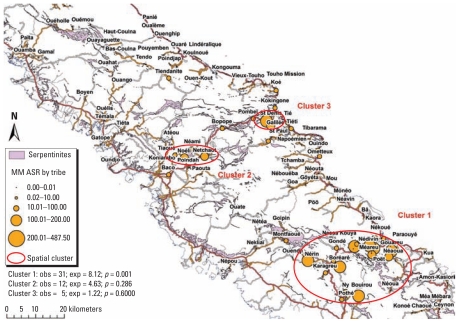
Map of the study area, MM incidence by tribe (residence 30 years before diagnosis), 1984–2008, and results of the spatial analysis. Abbreviations: obs, observed; exp, expected.

The spatial analysis highlighted one cluster grouping 18 central tribes in Houaïlou-Bourail area (Figure 2). Thirty-one cases were observed versus 8.12 expected cases, with a relative risk of 5.76 and a log-likelihood ratio of 23.02; *p* = 0.001. Two secondary clusters were observed in Koné and Poindimié, but these were not significant.

No significant temporal cluster was found. The spatiotemporal analysis showed one cluster grouping of 14 tribes in Houaïlou area for the 1991–2003 period: 18 observed cases, 3.10 expected cases, *p* = 0.001.

### Description of environmental determinants

#### The natural sources of asbestos

Based on geological maps and environmental sampling, we identified the following natural sources of asbestos: in the Boghen unit, serpentinite slivers were the host of tremolitic occurrences that, by weathering at the base of the soil horizon, evolve into white aggregates [Supplemental Material, Figure 2 (doi:10.1289/ehp. 1002862)]. This white soil was doughlike and similar to clay. In the peridotite massifs, three kinds of asbestos were observed: mainly antigorite having an appearance of friable rock, which splits up into smaller fragments, chrysotile occurring as small (in the millimeter to centimeter range) veins in fresh rocks, and sometimes tremolite [Supplemental Material, Figure 3 (doi:10.1289/ehp.1002862)]. The majority of asbestos occurrences in the Northern Caledonian Metamorphic complex were represented by tremolite-actinolite white aggregates.

We found three categories of fibers: short (< 20 μm) and thin (< 1 μm) tremolite fibers; long (> 100 μm) and flexible chrysotile fibers; and sheaves of fine fibers (< 1 μm), splintering from the edges of antigorite laths.

#### Serpentinite quarries

Within a 5-km radius around the center of each tribe, the surface of serpentinite outcrops exceeded 7 km^2^ in 12 tribes [Supplemental Material, Figure 4 (doi:10.1289/ehp.1002862)]. Forty-seven tribes were situated < 5 km from a serpentinite quarry. Seventeen tribes lived within a 20-km distance by road from > 10 serpentinite quarries.

#### Roads

Eighty-nine tribes lived < 10 km from the main road. The presence of serpentinite on roads (surface or road cut) was identified in 44 tribes. According to the local authorities, quarries and roads in our study area were in use at least at the end of the 1960s.

#### Whitewash of dwelling walls

The material used for whitewashing varied according to the geological environment of the tribe. Most of the tribes living near the sea used a soup made with fired coral. Others used white clay or crushed white rocks diluted with water, including silica and tremolite. Since the 1950s, the cob dwellings have been replaced with corrugated iron or concrete houses. The last year for whitewash use varied from 1950 to 2000. After 1970, fibrous Pö was still used in 41 tribes [Supplemental Material, Figures 5 and 6 (doi:10.1289/ehp.1002862)].

#### Mining activity

Fifty-five tribes lived < 10 km from the peridotite massif. The tonnage of nickel ore mined between 1940 and 1980 was substantial in the East of Houaïlou. About12,000,000 tons were extracted from the mines of Poro and 14,300,000 tons from the mines of Kouaoua [Supplemental Material, Figure 7 (doi:10.1289/ehp.1002862)].

#### Vegetation and situation

Four types of vegetation were observed in the environment of the tribes (data before 1996): bush, savannah, forest, and dense mining maquis. The plant cover was dense in 51 tribes (50 forest and 1 dense mining maquis) and nondense in 49 tribes (48 bush and one savannah).

The mean curvature was positive, indicating a convex shape, for only 22 tribes. Distance to the coastline was < 4 km for 51 tribes.

### Analyses of exposure and environmental factors

After grouping the smallest tribes, univariate and multivariate analyses were carried out on 82 geographical units.

#### Comparison of tribes with and without MM cases

The most significant risk factor highlighted by the univariate analyses (Table 1) is the presence of serpentinite on roads: OR = 465.0; 95% CI, 2.360–9161. This factor was present among 97% of tribes with MM cases versus 6% of tribes without MM cases (*p* < 0.001).

We also found significant increased risks (in descending order) for the presence of antigorite, surface of serpentinite outcrops, the number of serpentinite quarries in a 20-km radius, proximity to the peridotite, proximity to the nearest quarry of serpentinite, and presence of chrysotile. Proximity to the coast constituted a significantly protective factor.

Univariate Poisson regression (Table 1) showed similar results, with the highest IRR observed for serpentinite on roads (IRR = 60.654; 95% CI, 68.144–76.415). Statistically significant associations were also observed for the presence of antigorite, the distance to the peridotite massif, the number of serpentinite quarries, the surface area of serpentinite, the mine index, and the presence of chrysotile. Proximity to the coast line, dense vegetation, and mean slope >10° were inversely associated.

#### Interactions

The mine index was strongly correlated with the distance to the peridotite massif (*r* = 0.77; *p* < 0.0001). Thus, to avoid colinearity, we omitted the mine index in the multivariate analyses. Other correlations were ≤ 0.7.

Significant interactions were observed between the number of quarries and the presence of antigorite (*p* = 0.0159), the presence of chrysotile (*p* = 0.0025) and serpentinite on roads (*p* = 0.0026), and between the distance to the nearest quarry and serpentinite surface (*p* = 0.0218). These interactions were included in the multivariate logistic regression.

#### Multivariate analyses (Table 2)

Logistic regression resulted in one highly predictive factor: the presence of serpentinite on roads (OR = 465.0; 95% CI, 46.2–4679.7), which explained 72% of the total variance. We carried out a second logistic regression with the same variables except serpentinite on roads. This resulted in five predictive factors in the final model: presence of antigorite, distance to peridotite massif ≤ 10 km, serpentinite surface > 2 km^2^, presence of chrysotile, and negative curvature, which explained 52% of the total variance.

The multivariate Poisson regression model involved six environmental determinants: serpentinite on roads, antigorite, distance to peridotite massif ≤ 10 km, serpentinite surface > 2 km^2^, chrysotile, distance to nearest quarry ≤ 5 km, and three protective factors: mean slope < 10°, negative curvature, and distance to coast line ≤ 4 km, explaining 76% of the total variance.

## Discussion

Our study has confirmed the high risk of MM in New Caledonia and has identified geographical clusters. Living near roads covered by serpentinite was associated with the greatest risk of MM. Other significant risk factors included the number and the proximity of serpentinite quarries and the size of the surface area containing serpentinites.

Because of the good mechanical properties of serpentinite, its stability under rain, and the ease of crushing it, this material was widely used to cover roads, with the potential of asbestos exposure for those living closest to these roads. The large number of quarries along the road between Koné and Poindimié provides evidence of this extensive practice until the completion of the present sealed road. Serpentinite quarries are numerous in the Houaïlou area, and some of them were still in operation during our investigations.

The second interesting result is the difference in risk between serpentines and amphiboles. In particular, a much stronger association with MM risk was found for antigorite (OR = 20.667; 95% CI, 4.028–106.03) compared with tremolite-actinolite (OR = 1.714; 95% CI, 0.664–4.423). This difference may be explained by the shape or the use of these minerals. Tremolite commonly comes in the form of doughy and aggregated white soil under the vegetable layer, whereas the fibrous form of antigorite comes from the weathering of the rocks, which produces increasingly small pieces down to very fine dust.

Unofficial serpentinite quarries containing mostly antigorite were abundant, and this material was very commonly used to cover the roads. The two serpentines chrysotile and antigorite have very similar chemical composition but different structures. Antigorite presents lathes that splinter, whereas chrysotile has a coiled structure. Recent works showed the cytotoxicity of antigorite fibers *in vivo* (Pugnaloni et al. 2010). However, antigorite does not belong to industrial asbestos and is therefore not recognized in asbestos regulation.

In the 1930s, Melanesians were encouraged to whitewash the walls of their dwellings. This Pö could be made with different material, depending on the environment. Tremolite was used by melting this paste with hot water. This practice was widespread until the 1990s in areas where MM incidence rates were low; census data showed 95 tremolitic dwellings in Poya (three MM cases, two living in tribes) and 65 in Hienghène (one MM case) compared with only 26 dwellings in Houaïlou and 17 in Poindimié, where the MM incidences were the highest. Consistent with this, the use of whitewash did not emerge as a significant risk factor for MM in our study. The different indicators (number of tremolitic dwellings, use of tremolitic Pö after 1970, last year of Pö use) confirm this result and contradict previous studies (Luce et al. 2000). Thus, either natural tremolite fibers are less hazardous, or tremolite aggregates as used in whitewash retain the fibers and avoid their dispersal and subsequent exposure.

Mining activity produces dust that may contain asbestos fibers when peridotites are serpentinized. This dust can affect tribes downwind from the mines. However, our mine index showed only a very small effect; proximity to the peridotite massifs, on the other hand, was significantly associated with MM. Living on a slope > 10° and around dense vegetation (nonsignificant) appeared to be protective, probably because they prevent dust propagation.

Environmental exposure is usually related to low relative risks with a complex mixture of several low-level intercorrelated exposures (Pekkanen and Pearce 2001). Because of the long latency period and the involuntary nature of environmental exposure, it is difficult to reconstruct personal exposure accurately. Most environmental variables, such as presence of serpentinite on roads, mining activity, and plant cover, are measured at the population level, so they can be analyzed by comparing populations rather than individuals. We therefore chose an ecological study design to assess the association between environmental asbestos exposure and MM. To minimize the typical problems associated with ecological analyses, and because our environmental data are relative to the tribes, we studied spatial units at the tribal level. Environmental data were similar to only nearby tribes, which were grouped when the population of one tribe was very small.

To test whether some MM cases were not identified by the cancer registry, we checked all MM cases recorded in the death certificate files, questioned the public and private lung specialists, and visited the health centers. We did not find any new cases. We also studied the geographic distribution of all cancer incidence in New Caledonia for 1990–2005 (results not shown). The ASR of all cancers was in the same order in Hienghène and Poya as in the other communes. Hienghène showed the highest incidence rate among women (due to very high incidence of thyroid and breast cancer), indicating good quality of medical detection and registration of cancers in this area. Thus, the low MM incidence in Hienghène and Poya is unlikely to have arisen from a lack of registration, and the observed spatial MM distribution is unlikely to be due to selection effect.

Information on occupation was insufficient to allow the role of occupational exposure to be assessed. This is a limitation of our study. However, the sex ratio (M:F < 1) of MM cases and the mean age of < 60 years for both sexes suggest a high probability of a causal environmental exposure rather than occupational exposure. Moreover, occupational sources of exposure are unlikely to follow the same spatial distribution as environmental sources. Therefore, residual confounding by occupational exposure is not likely to explain our results.

Pan et al. (2005) showed an inverse association between mesothelioma and the distance that people lived from the nearest naturally occurring asbestos in California. Our study adds to the evidence that natural sources of asbestos increase the risk of mesothelioma, and a strength of our study is that it takes into account multiple exposure sources and protective environmental factors.

## Conclusions

This study provides strong evidence that the use of serpentinite for road surfacing is a major environmental risk factor for mesothelioma in New Caledonia. We communicated our first results to health and local authorities, which resulted in the government establishing inventories of quarries and sites built on naturally occurring asbestos and in initiating rehabilitation studies and new laws to protect workers. The same measures have not yet been established for residents.

Most environmental asbestos fibers are short and thin. As for antigorite fibers, short fibers are not usually taken into account in analyses. Recent studies have shown that these fibers could be responsible for mesothelioma (Suzuki et al. 2005). Thus, to understand the hazards of natural sources of asbestos, studies are now needed on the ability of these sources to release fibers into the air, and the types and sizes of fibers causally related to the disease.

## Figures and Tables

**Table 1:** Univariate analyses of environmental factors influences on MM ASR by tribe.

Univariate analyses/Variables	Comparison of tribes with MM (*n* = 34) versus tribes without MM cases (*n* = 48)		Comparison of mean MM ASR by covariate class		
	No. of tribes	OR (95% CI)	MM ASR	*p*-Value	Poisson IRR (95% CI)
Serpentinite on track					
Absence	46	1	1.6		
Presence	34	465.00 (2.360–9,161)	97.0	< 0.0001^a^	60.654 (68.144–76.415)

Antigorite					
Absence	35	1	3. 3		
Presence	47	20.667 (4.028–106.03)	71.2	< 0.0001^a^	21.682 (18.003–26.114)

Serpentinite surface (km^2^)					
0–1.2	27	1	3.2		
1.21–3	31	13.333 (2.683–66.256)	52.4		
3.01–30	24	25.000 (4.698–133.03)	72.9	0.0001^a^	2.484 (2.366–2.609)

No. of quarries < 20 km					
0–3	32	1	8.9		
4–6	27	4.320 (1.276–14.623)	44.0		
7–21	23	15.300 (4.034–58.021)	86.4	0.0004^b^	2.665 (2.541–2.795)

Distance to massif (km)					
10.01–43	39	1	7.9		
0–10	43	7.716 (2.423–24.570)	73.3	< 0.0001^a^	9.309 (8.281–10.466)

Distance to quarry (km)					
9.1–31	25	1	29.9		
3.1–9	29	2.550(0.875–7.429)	39.9		
0–3	28	9.452 (2.526–35.361)	55.6	0.0265^b^	1.368 (1.310–1.427)

Chrysotile					
Absence	54	1	29.164		
Presence	28	4.275 (1.520–12.023)	67.339	0.0289	2.309 (2.159–2.469)

Coast distance (km)					
4.1–22	35	1	60.4		
0–4	47	0.318 (0.122–0.830)	28.7	NS	0.464 (0.444–0.508)

Curvature (in degrees)					
> 0	16	1	55.0		
≤ 0	66	0.343 (0.107–1.096)	39.1	NS	0.711 (0.659–0.768)

No. of censed dwellings					
0	37	1	28.9		
1–54	45	2.471 (0.989–6.174)	53.1	NS	1.834 (1.706–1.971)

Dense vegetation					
No	38	1	58.0		
Yes	44	0.420 (0.166–1.059)	28.5	NS^a^	0.492 (0.456–0.527)

Distance to main road (km)					
1.1–36.5	43	1	39.6		
0–1	39	2.180 (0.871–5.458)	45.0	NS	1.136 (1.062–1.214)

Mine index (tonne/m)					
0	47	1	26.5		
> 0	35	2.051 (0.821–5.126)	63.3	0.0438^a^	2.392 (2.232–2.564)

Tremolite-actinolite					
Absence	30	1	38.7		
Presence	52	1.714 (0.664–4.423)	44.2	NS	1.142 (1.065–1.226)

Mean slope (in degrees)					
10.1–24.6	50	1	51.0		
1.6–10	32	1.167 (0.472–2.884)	28.4	NS	0.557 (0.517–0.601)

Whitewashing					
Nonfibrous	18	1	43.9		
Partly fibrous	16	1.333 (0.337–5.270)	88.5		
Totally fibrous	48	0.330 (0.108–1.008)	26.1	0.0451	0.727 (0.670–0.755)

No. of years of Pö use after 1945					
0–10	28	1	48.3		
10.5–30	28	1.000 (0.351–2.851)	57.2		
30.5–55	26	0.300 (0.093–0.972)	19.5	NS^b^	0.709 (0.680–0.740)

Abbreviations: ASR, world age-standardized incidence rate per 100,000 person-years; NS, nonsignificant.

a Welch test.

b Kruskal-Wallis test.

**Table 2:** Multivariate analyses of environmental factors influences on MM ASR by tribe.

Tribes with MM (*n* = 34) versus tribes without MM cases (*n* = 48)	Logistic regression (pseudo *R*^2^ = 0.7221)	
Variable	OR (95% CI)	*p*-Value
Serpentinite on roads: presence/absence	465.0 46.2–4679.7	< 0.001
Tribes with MM (*n* = 34) versus tribes without MM cases (*n* = 48)	Logistic regression without serpentinite on roads (pseudo *R*^2^ = 0.5334)	
Variable	OR (95% CI)	*p*-Value

Antigorite: presence/absence	35.823 (4.053–316.6)	0.001
Distance to massif: 0–10 km/> 10 km	19.825 (2.935–134.05)	0.002
Serpentinite surface: 2.01–30/0–2 km^2^	9.773 (1.871–51.04)	0.007
Chrysotile: presence/absence	9.652 (1.638–56.860)	0.012
Curvature: ≤ 0/> 0	0.157 (0.0074–0.503)	0.009
Multivariate analysis of MM ASR (*n* = 80)	Poisson regression (*R*^2^ = 0.7615)	
Variable	IRR (95% CI)	*p*-Value

Serpentinite on roads: presence/absence	13.052 (10.225–16.661)	< 0.001
Antigorite: presence/absence	4.799 (3.880–5.935)	< 0.001
Distance to massif: 0–10 km/> 10 km	3.241 (2.812–3.736)	< 0.001
Serpentinite surface: 2.01–30/0–2 km^2^	2.430 (2.158–2.728)	< 0.001
Chrysotile: presence/absence	1.638 (1.547–1.832)	< 0.001
Distance to quarry: 0–5 km/> 5 km	1.205 (1.104–1.315)	< 0.001
Mean slope: > 10°/< 10°	0.679 (0.599–0.701)	< 0.001
Curvature: ≤ 0/> 0	0.844 (0.775–0.919)	< 0.001
Coast distance: 0–4 km/> 4 km	0.849 (0.784–0.818)	< 0.001
